# Application of principal component analysis in the pollution assessment with heavy metals of vegetable food chain in the old mining areas

**DOI:** 10.1186/1752-153X-6-156

**Published:** 2012-12-13

**Authors:** Iosif Gergen, Monica Harmanescu

**Affiliations:** 1 Banat’s University of Agricultural Science and Veterinary Medicine Timisoara, Faculty of Food Processing Technology, Calea Aradului 119, RO, 300645, Romania; 2 Banat’s University of Agricultural Science and Veterinary Medicine Timisoara, Faculty of Agriculture, Calea Aradului 119, RO, 300645, Romania

**Keywords:** Heavy metals, Soils, Vegetables, Long-lasting mining activities, PCA

## Abstract

**Background:**

The aim of the paper is to assess by the principal components analysis (PCA) the heavy metal contamination of soil and vegetables widely used as food for people who live in areas contaminated by heavy metals (HMs) due to long-lasting mining activities. This chemometric technique allowed us to select the best model for determining the risk of HMs on the food chain as well as on people's health.

**Results:**

Many PCA models were computed with different variables: heavy metals contents and some agro-chemical parameters which characterize the soil samples from contaminated and uncontaminated areas, HMs contents of different types of vegetables grown and consumed in these areas, and the complex parameter target hazard quotients (THQ). Results were discussed in terms of principal component analysis.

**Conclusion:**

There were two major benefits in processing the data PCA: firstly, it helped in optimizing the number and type of data that are best in rendering the HMs contamination of the soil and vegetables. Secondly, it was valuable for selecting the vegetable species which present the highest/minimum risk of a negative impact on the food chain and human health.

## Background

Since ancient times, plant based food has played an important role in human nutrition, being a very important source of antioxidants, vitamins and minerals [1-3]. Modern nutrition requires greater consumption of vegetables and fruits, because of their role on the quality of life [1]. On the other hand, plant food, especially the one which is consumed without prior processing, such as raw vegetables, is the first link of the food chain through which macro and micro metals can go directly into the body. To ensure the functioning of various enzyme systems, the human body needs most micro metals, but some such as Cd, Pb, and Hg have toxic effects. Toxicology indicates that other heavy metals, such as Cu, Ni, Mn, Fe, Cr, V, Mo, can appear in the list of harmful metals, if their concentration exceeds certain limits [4,5].

For vegetables obtained in uncontaminated areas, the levels of these metals are low, generally below the permissible limits. The situation is quite different when these crops are obtained in geogenic or anthropogenic contaminated areas, such as the mining areas where these metals are exploited. Especially after the industrial revolution, the need for metals in modern society led to the development of metal mining. Because environmental pollution problems have generally been neglected over the centuries, accumulated residues in these important areas have led to increased pollution of soil and plants (both spontaneous and cultivated). Only in recent decades has society realized the negative effects that heavy metals contamination has on the environment and on human health. Nowadays, heavy metals have been acknowledged as factors of “Global Change” scenarios. Climate change may affect HMs bioavailability in soils and hence the entire food chain [6].

Romania is recognized as one of the European countries with polymetallic mining (Fe, Pb, Cu, Zn, Au, Ag) since antiquity [7]. Research conducted in Romania in recent decades has clearly shown the extent of the anthropogenic pollution of soil and plant food (both of these mining areas as well as of the large urban areas) with various heavy metals [8-13]. Such research, which calls attention to the increased contamination of soils and plants in areas contaminated by mining activities or in large conurbation areas, has been performed worldwide [14-18].

Together with soil, vegetable food constitutes the next link that can increase HMs accumulation in the food chain and that can make them a hazard for consumption. Recently, many studies on various plant foods have addressed this issue [19-32]. If Fe, Mn, Zn, Cu, Ni are essential for plant growth, other HMs (Cd, Pb, Cr, Hg, Ag) are toxic. In normal situations, plants' defences and homeostasis by complex mechanisms are processes that limit the accumulation of HMs [33], for example in special situations in contaminated areas where HMs accumulation in some vegetables can be high and dangerous for human health [19,30-32].

For proper growth and development of all animals, including humans, Fe, Mn, Zn and Cu can be considered trace minerals with a central role in many metabolic processes throughout the body. They are essential as catalysts in many enzyme and hormone systems which influence growth, bone development, feathering, enzyme structure and function. On the other hand, it has been proven that, in large amounts, these metals can cause oxidative stress in the animal body, which can on the one hand be beneficial, killing tumour cells, but on the other hand it may have a negative role inducing cancer by oxidative DNA lesion [34]. Moreover, science has proven the existence of structural interactions between heavy metals and functional peptides [35-37]. Another source of metals in animal-based food for humans is the addition of metals in forage, which can get into meat or other animal food products and hence in the human body, where it can influence human health in a positive or negative way [38-40].

The wide range of aspects regarding the presence of HMs in the food chain and their implications on human health requires further research in this field in a unitary approach on the environment which the humankind is part of. The old mining areas affected by anthropogenic contamination with heavy metals are particular areas in which the concentration of one or more heavy metals exceeds normal values in most soils and in some agricultural products used as plant food, such as vegetables and fruit, or even animal products (meat, eggs, and milk).

Given the variety and diversity of data, research in these areas involves not only modern analytical methods with sensitivity, specificity and high accuracy to obtain valid results on HMs content in soil and food but also complex statistical methods that provide the big picture in what they are concerned. Multivariate statistical techniques are the right tool for viewing and analysing some matrices of complex data [41]. PCA and cluster analysis (CA) are two unsupervised methods that allow us to deduce how certain variables (metals concentration, other parameters of the soil or plants) that characterize objects (soil, plant) determine their association. If the CA method is used for samples grouping original variables, PCA estimates the correlation structure of the variables by finding hypothetical new variables (principal components - PC) that account for as much as possible of the variance (or correlation) in a multidimensional data set. These new variables are linear combinations of the original variables [42]. This method helps us to identify groups of variables (i.e. heavy metals concentrations or other soil or plant parameters) based on the loadings and groups of samples (soil or vegetable species) based on the scores.

To understand the complex connection between soil or plant samples and heavy metals contents was used chemometric technique PCA. It is based on eigenanalysis of the covariance or correlation matrix. Each variable has a loading which show how well a variable is taken into account by the model components. They reflect how much each variable contributes to the meaningful variation (or correlation) in the data and to interpret variables relationship. Each sample has a score along each model component which shows the location of the sample in this model and can be used to detect sample patterns, groupings, similarities or differences [43,44]. In practice, it will ignore higher number PC axes that explain only a small proportion of variance in the species data [45]. The importance of a variable in a PC model is indicated by the size of its residual variance. This is useful for the variable selection; a variable with little explained variance may be removed without adding more changes to the PC model. It is not restriction in the number of variables, the rule for multiple regressions that the number of variables must be smaller than the number of objects does not apply in PCA case. The closer the similarity between the objects, the fewer terms are needed in the expansion to achieve certain approximation goodness [46]. In order to simplify plotting, PCA may be used for reduction of the data set to only two variables (the first two components).

In recent years, chemometric evaluation has increasingly been used in food research. Most applications of chemometric methods focus on establishing correlations between different foods and their composition [41,44,47-49], determining geographical origin [50-52], quality of water from dew [53], quality of environment [54], modelling of heavy metals contamination of fruits and vegetables [55,56] or authentication of organic food [57].

The purpose of our work is to assess the complex phenomenon of pollution of the vegetable food chain in old mining areas with heavy metals by the Principal Component Analysis. For this purpose, many PCA models were constructed and they were computed with different markers. As main markers for pollution were used two types of markers: simple markers, represented by the HMs concentrations in contaminated soils and by the HMs concentrations in the vegetables consumed by the population in these areas. It was also employed a complex marker, namely Target Hazard Quotiens (THQ). This marker connects the metals concentrations in food with their toxicity, quantity and quality of food consumption and body mass of consumers [58]. The use of this complex parameter is more extensive in evaluating the potential health risk of HMs present in various foods [58-62].

## Results and discussion

### Assessment of soil data by principal component analysis

Soil is the main link in the food chain. From soil, vegetables can take both nutrients and toxic elements, such as heavy metals, directly by root adsorption and indirectly, through foliar absorption of contaminated soil particles. Extensive exploitation of metals by mining left traces in the soils of these areas (R and M), compared with the reference area (Ref) in which there were no mining activities. The levels of heavy metals found in soil samples from the three areas cultivated with different crops of vegetables are presented in Table 1. All these data were selected as variables for Principal Component Analysis and computed the PCA1 - soil model.

**Table 1:** **Levels of heavy metals in soils under vegetables from contaminated and reference areas (mgkg**^**-1**^**, average values** ±SDV,* p < 0.05)

**Samples**	**Code**	**Fe**	**Mn**	**Zn**	**Cu**	**Ni**	**Cd**	**Pb**
Root parsley R	R1	75833*±2572	12268*±1416	449*±37	70*±20	16.96*±4.15	1.63*±0.56	110.12*±12.41
Root parsley M	M1	45713*±916	1870±239	200*±23	238±31	18.04*±1.23	0.41*±0.07	20.10±2.39
Root parsley Ref	Ref1	28896±851	3886±260	97±15	19±5	9.95±0.58	0.11±0.01	15.69±2.30
Root carrot R	R2	54138*±1512	3833*±731	268*±28	42*±5	14.96*±1.90	2.19*±1.48	91.95*±10.75
Root carrot M	M2	45687*±774	1994±237	204*±20	230*±27	18.39*±1.10	0.42*±0.06	20.43±2.05
Root carrot Ref	Ref2	32492±1827	2414±678	131±19	26±16	10.34±0.89	0.17±0.04	16.13±1.58
Onion R	R3	51667*±3193	6537*±1473	367*±47	50*±9	15.13±1.90	1.85*±0.60	143.80*±7.58
Onion M	M3	44656*±801	1572±214	178±28	201*±32	18.17±1.73	0.34*±0.08	24.17*±3.62
Onion Ref	Ref3	33725±1498	2437±668	150±17	26±4	11.63±1.28	0.19±0.03	16.14±2.39
Leaf parsley R	R4	56020±1438	7021*±1107	406*±59	46*±11	15.67*±2.41	2.25*±0.79	123.32*±9.12
Leaf parsley M	M4	44046±1693	1870*±239	200*±23	238*±31	18.04*±1.23	0.41±0.07	21.77*±2.08
Leaf parsley Ref	Ref4	32302±2694	3063±493	107±19	18±4	9.27±0.79	0.12±0.01	13.76±2.60
Leaf carrot R	R5	54138*±1511	3833±731	301*±60	42*±5	14.96*±1.90	2.19*±1.48	161.95*±6.15
Leaf carrot M	M5	44046*±1693	1869±239	200*±23	238*±31	18.04*±1.23	0.41*±0.07	21.77±2.08
Leaf carrot Ref	Ref5	32492±1827	2414±678	131±19	21±3	10.34±0.89	0.17±0.04	15.46±2.05
Cabbage R	R6	53623*±2876	4336±664	349*±47	54±10	14.30±1.30	2.11*±0.81	119.19*±11.70
Cabbage M	M6	45488*±1088	1962±269	211±25	254*±32	18.73*±1.25	0.42*±0.09	23.35±2.03
Cabbage Ref	Ref6	34511±1562	2847±578	148±20	26±4	11.86±1.55	0.20±0.04	17.89±2.00
Lettuce R	R7	55173*±2047	5478*±617	367*±42	47±8	13.24±0.51	2.32*±0.60	130.97*±13.38
Lettuce M	M7	44730±1047	2077*±417	114±22	60*±1	22.79±.51	0.31±0.40	45.27±7.38
Lettuce Ref	Ref7	34270±612	344±120	171±9	31.44±5.89	13.47±2.19	0.19±0.02	20.00±2.72
Cucumber R	R8	55925*±1101	5521*±844	363±52	41±6	13.18±0.61	2.46*±0.86	126.57*±12.14
Cucumber M	M8	44046*±1693	1869±239	200±23	238*±31	18.04*±1.23	0.41*±0.07	21.77±2.08
Cucumber Ref	Ref8	31825±2067	890±112	138±13	25±4	11.47±1.30	0.18±0.02	17.69±1.73
Green Bean R	R9	51653*±3264	3774±669	307±66	38*±5	13.89±0.74	1.11*±0.38	119.69*±8.96
Green Bean M	M9	42156±1803	1572*±213	178±28	201*±32	18.17*±1.73	0.34*±0.08	19.17±1.87
Green Bean Ref	Ref9	32792±1573	3089±320	143±16	24±4	11.22±1.16	0.18±0.03	16.60±2.07

Compared with normal contents and alert values, in accordance with the Romanian legislation (Table 2), the most frequent and pronounced excess of normal values was observed for Mn followed by Zn, Cu and Pb. For both Ni and Cd, no excess of the legally admissible contents was found in contaminated areas or in the reference area.

**Table 2:** **Normal contents, threshold and intervention threshold values for heavy metals in Romanian legislation (mgkg**^**-1**^**)**

**Soil samples under**	**Fe**	**Mn**	**Zn**	**Cu**	**Ni**	**Cd**	**Pb**
Normal contents (NC)	**-**	900	100	20	20	1	20
Alert threshold values (ATV)	**-**	1500	300	100	75	3	50
Intervention threshold values (ITV)	**-**	2500	600	200	150	5	100

**Figure 1:**
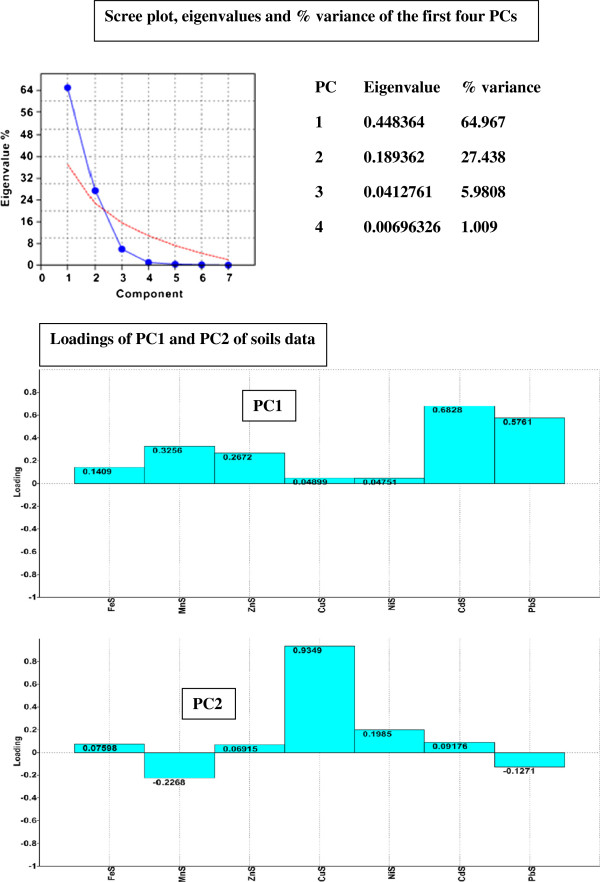
**Scree plot and PC loadings of soils data, in PCA1-soil model.** (FeS, MnS, ZnS, CuS, NiS CdS, PbS are variables used in PCA1-soil model that symbolized total contents of Fe, Mn, Zn, Cu, Ni, Cd and Pb in soil samples).

**Figure 2:**
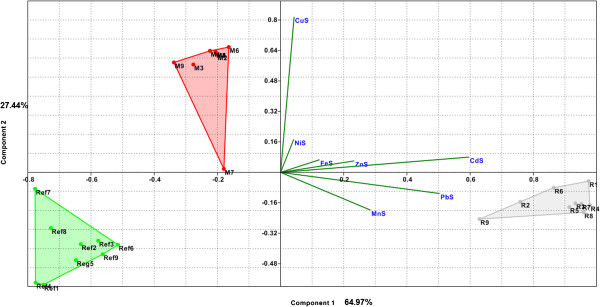
**Biplot of PC1 and PC2 for soil data in PCA1-soil model.** (FeS, MnS, ZnS, CuS, NiS, CdS and PbS are variables used in PCA1-soil model that symbolized total content of Fe, Mn, Zn, Cu, Ni, Cd and Pb in soil samples; R, M and Ref symbolized soils samples from contaminated respectively reference areas and numbers 1 to 9 attached to previous symbols refer to soils samples associated with vegetables species or edible part: 1 for parsley roots, 2 for carrot roots, 3 for onion bulbs, 4 for parsley leaf, 5 for carrot leaf, 6 for cabbage, 7 for lettuce, 8 for cucumber and 9 for green bean).

To understand the association of soil samples from the three areas, depending on heavy metals content, Principal Components Analysis was applied using PAST software [42]. In the PAST software, the PCA routine finds the eigenvalues and eigenvectors of the variance-covariance (var-covar) matrix or the correlation matrix. Var-covar is used if all variables are measured in the same units (e.g. concentration in mg kg^-1^). Correlation (normalized var-covar) is used if the variables are measured in different units (e.g. metal concentration in mg kg^-1^, clay in % and pH in pH units); this implies normalizing all variables using division by their standard deviations. The eigenvalues give a measure of the variance accounted by the corresponding eigenvectors (components) [42]. Given the large scale of values for metal concentrations (from unit to thousand), to standardize the data, we performed logarithmic data transformation. From scree plot graph of eigenvalues of the PCA1-soil model (Figure 1) it can be seen that the first two PCs are enough to explain 94.2% of the pattern variation. Concentrations in Cd, Pb and Zn were major contributors to PC1 while the Cu concentration was the major contributor to PC2. The two factors can separate well the two areas (R and M) with anthropogenic pollution caused by mining, one from each other and both from the reference (Ref) unpolluted area (Figure 2). The Cu concentration is mainly responsible for separating the M area; this variable is the one with the highest positive loadings on PC2 (Figure 1). This area is well-known for copper exploitation, and the concentration of copper in the investigated soils generally exceeds ATV values (Table 1). PC1 contributes most significantly for the separation of R area, which is known for its deposits of polyminerals, especially Pb. Along Pb, Cd and Zn there are some other metals with a significant contribution on PC1 (Figure 1).

In the R area these metals have concentrations that exceed the permissible amount of ATV (for Pb and Zn) or get very close to it (for Cd). Fe, Mn and Ni are metals that in terms of geochemical investigation are common for the three areas and also show reduced contribution of the two PC components.

PCA can be seen as an ordination technique that constructs the theoretical variable that minimizes the total residual sum of squares after fitting a straight line to the data for each species. PCA does so by choosing best values for the site, i.e. the site scores. A positive score means that the concentration of variables increases along the PC axis; a negative score means that the concentration of variables decreases along the axis and a score near 0 means that the concentration is poorly (linearly) related to the PC axis. The direction of the variable arrows indicates the direction in which the concentration of the corresponding species increases most, and the length of the arrows equals the rate of change in that direction. In the perpendicular direction, the fitted concentration is constant [45]. Soil samples from R area, with old polymetallic exploitation (especially Pb), are grouped in the positive side of PC1 and closer to the variables Cd, Pb which present the highest concentrations in all samples from this area. Soil samples from M area, known for Cu exploitation, are grouped in the positive side of PC2, and mainly consist of variable Cu (Figure 2) which presents the highest concentrations in these samples. Soil samples from the Ref area, with the lowest content of heavy metals, are grouped in the negative side of the two PCs, revealing their reduced influence on the reference sample group. Based on these considerations, can be eliminated the variables Fe, Mn and Ni from the model without affecting its quality. For the new PCA2-soil model (see Additional file 1), with only 4 variables (Cu, Zn, Pb and Cd), the two factors explain 98.6% of model variance, which means that variables that were eliminated had the role of noise, contributing to decreased quality of the model. Thus, by using PCA, was obtained a reduction of the number of analyses from 7 to 4 (and also the analytical cost) needed to correctly characterize and classify the soil samples from the areas under research, in terms of pollution with toxic or potentially toxic heavy metals.

### Assessment of the vegetables-related data by principal component analysis

To characterize by PCA the heavy metals contamination of vegetables in the afore-mentioned areas, according to location (contaminated or uncontaminated areas), first was constructed a PCA1 - plant model, containing only the concentration of HMs in vegetables (Table 3). The quality of the model was poor in vegetable samples classification according to location (see Additional file 2). To improve the model, in addition to analytical data of heavy metals concentrations in plants, was also used some agrochemical soil characteristics, because the translocation of metals in plants is dependent on the agrochemical characteristics of soil, such as pH and clay content [10,12].

**Table 3:** **Levels of heavy metals in vegetables from contaminated and reference areas (average values in mgkg**^**-1**^**, fresh matter) adapted after Harmanescu et al., 2011**

**Vegetables and location/Metals**	**Code**	**Fe**	**Mn**	**Zn**	**Cu**	**Ni**	**Cd**	**Pb**
Root parsley R	R1	221.80	37.83	45.82	6.88	1.28	0.20	15.78
Root parsley M	M1	99.02	8.10	7.74	6.66	0.49	0.04	0.66
Root parsley Ref	Ref1	48.78	3.27	3.52	1.22	0.19	0.01	0.08
Root carrot R	R2	29.97	3.06	4.93	1.77	0.18	0.08	2.11
Root carrot M	M2	31.89	2.22	3.18	1.54	0.08	0.03	0.09
Root carrot Ref	Ref2	17.31	1.43	2.05	0.74	0.04	0.01	0.06
Onion R	R3	15.26	4.07	10.91	1.37	0.21	0.06	0.50
Onion M	M3	1.56	0.32	0.78	0.25	0.01	0.01	0.03
Onion Ref	Ref3	4.65	1.34	2.01	0.43	0.03	0.01	0.04
Leaf parsley R	R4	73.17	5.88	9.39	1.77	0.30	0.09	1.97
Leaf parsley M	M4	106.75	9.72	10.44	4.79	1.87	0.05	0.50
Leaf parsley Ref	Ref4	104.03	7.32	9.13	1.03	0.38	0.03	0.28
Leaf carrot R	R5	14.25	1.38	1.36	0.29	0.11	0.01	0.20
Leaf carrot M	M5	51.45	5.57	5.61	2.12	0.38	0.04	0.12
Leaf carrot Ref	Ref5	31.59	3.13	3.77	0.40	0.17	0.01	0.03
Cabbage R	R6	60.11	10.47	16.30	1.36	0.70	0.12	0.90
Cabbage M	M6	31.53	9.15	8.51	2.77	0.33	0.06	0.25
Cabbage Ref	Ref6	16.06	3.85	3.28	0.45	0.13	0.01	0.05
Lettuce R	R7	35.88	8.33	14.46	1.86	0.28	0.09	0.62
Lettuce M	M7	13.60	4.12	5.14	2.22	0.18	0.09	0.21
Lettuce Ref	Ref7	16.90	3.46	5.32	0.76	0.10	0.02	0.08
Cucumber R	R8	2.39	6.73	8.96	0.98	0.28	0.13	0.72
Cucumber M	M8	2.38	6.01	1.39	2.42	0.54	0.15	0.37
Cucumber Ref	Ref8	1.70	0.45	0.95	0.45	0.23	0.03	0.16
Green Bean R	R9	58.85	7.06	13.22	1.05	0.49	0.06	0.35
Green Bean M	M9	21.65	4.77	10.17	1.45	0.52	0.07	0.19
Green Bean Ref	Ref9	26.19	6.45	9.68	0.29	0.22	0.03	0.07

**Figure 3:**
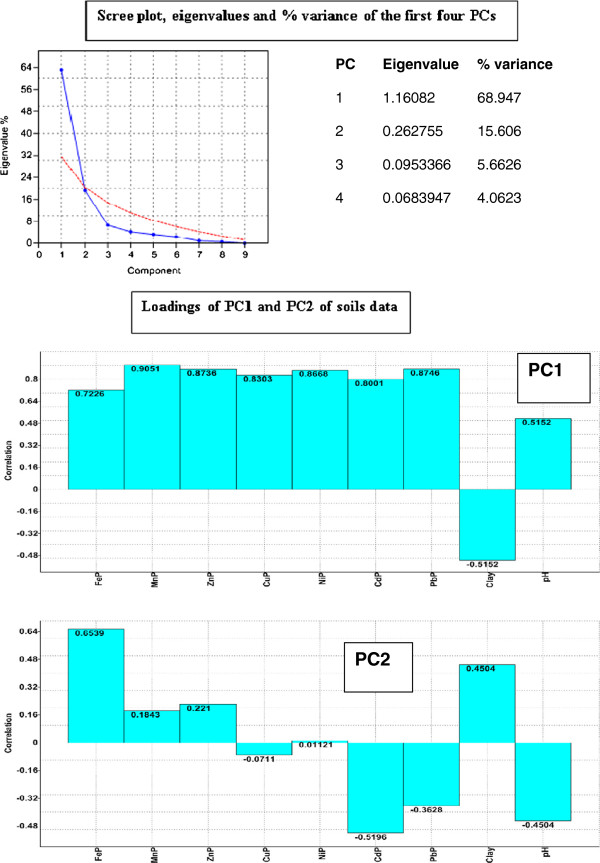
**Scree plot and PC loadings of vegetables data in PCA2-plant model.** (FeP, MnP, ZnP, CuP, NiP, CdP and PbP are variables used in PCA2 –plant model that symbolized total contents of Fe, Mn, Zn, Cu, Ni, Cd and Pb in vegetable samples, reported to fresh matter; clay and pH are agrochemical soil parameters used in this model).

**Figure 4:**
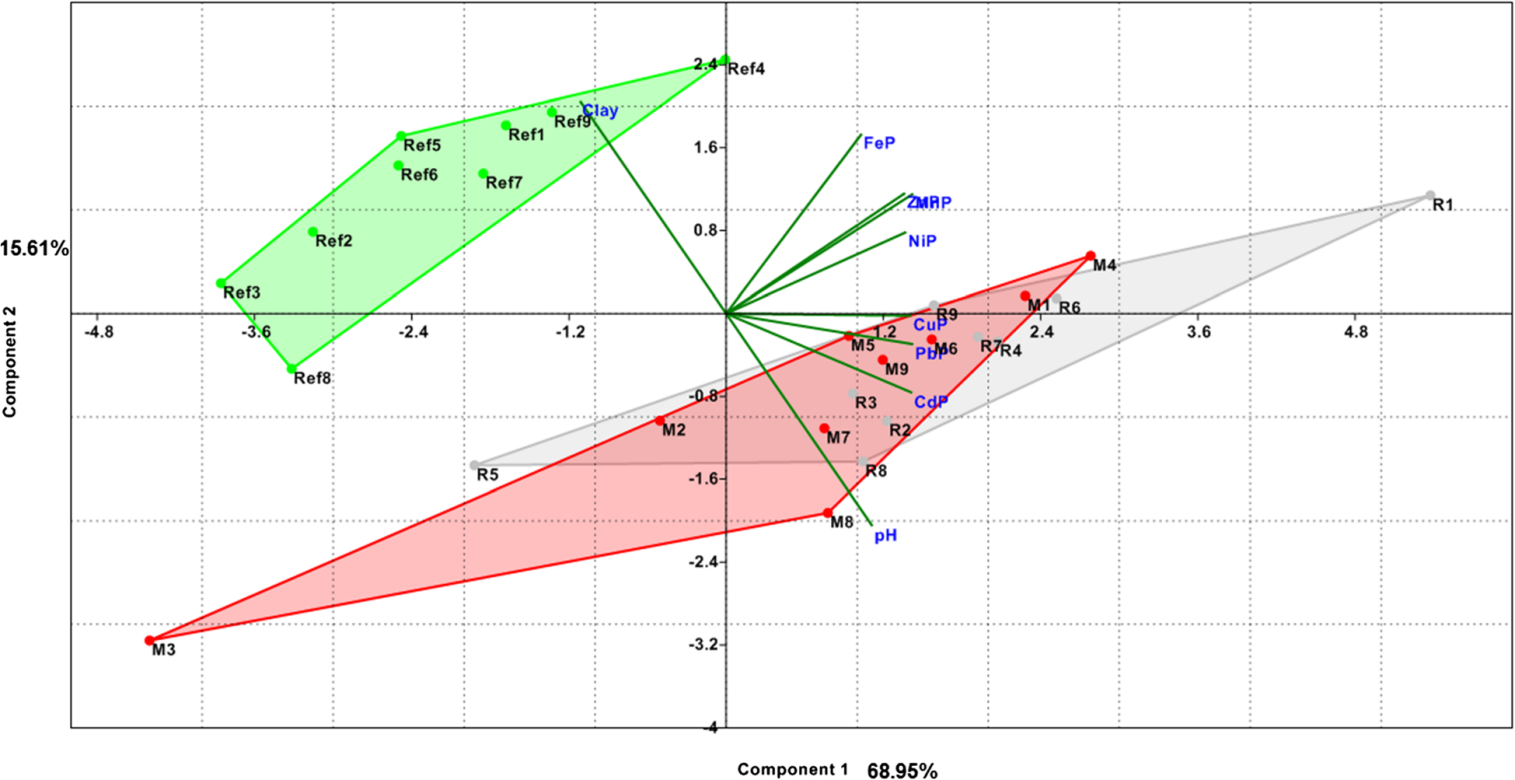
**Biplot of PC1 and PC2 for vegetable data grouped on location, used PCA2-plant model.** (FeP, MnP, ZnP, CuP, NiP, CdP and PbP are variables used in PCA2-plant model that symbolized total contents of Fe, Mn, Zn, Cu, Ni, Cd and Pb in vegetable samples, reported to fresh matter; clay and pH are agrochemical soil parameters used in this model; R, M and Ref symbolized vegetables samples from contaminated respectively reference areas and numbers 1 to 9 attached to previous symbols refer to vegetables species or edible part: 1 for parsley roots, 2 for carrot roots, 3 for onion bulbs, 4 for parsley leaf, 5 for carrot leaf, 6 for cabbage, 7 for lettuce, 8 for cucumber and 9 for green bean).

Similarly with the data related to soil, the vegetables-related data were standardized by logarithms and processed with the same PAST software. Scree plot and PC loadings for this PCA2 - plant model are presented in Figure 3. One can see that, in this model, the first two PCs explained only 84.4% of the variance. Graphical representation of the PCA2 - plant model, biplot of PC1 and PC2 (Figure 4), distinctly separate only areas with anthropogenic pollution of the soil (R, M) from the unpolluted area (Ref). This model, built on the metals content of plants and the two agrochemical parameters, cannot make a clear differentiation between the two areas with anthropogenic pollution (R and M). In the same way, no other combination of PC1 and PC3 and PC4, with a lesser degree for variance explanation, provides good differentiation between these three areas.

In the reference area, although slightly-acid pH is favourable for the mobility of heavy metals, high clay content of the soil reduces their translocation in plants. PC2 and its components are the factors that differentiate this area from the other two (M and R), which are characterized mainly by PC1 and its components (Figure 4).

Fe, Mn, Zn and Ni are the metals found in high concentrations in vegetables in the reference area. In the vegetables from the areas with anthropogenic contamination (R and M), Pb, Cu and Cd have the highest concentrations; they also present the highest concentrations in the soils from these areas. These metals are strongly absorbed from this low-clay soil, although its neutral pH is less favourable for the mobility of metals [10,12]. Similar agrochemical characteristics of the soils in areas contaminated with heavy metals (pH, humus and clay) as well as the high heavy metals contents of these soils, account for the similarity in their make their translocation in plants. The significant differences found between the Cu and Pb concentrations in the soils of the two contaminated areas (M and R) were not also found in the vegetables grown in the two areas. This can be explained by the homeostasis of plants, which, by mechanisms that are specific for each species, limits excessive accumulation of heavy metals in their bodies [33].

**Figure 5:**
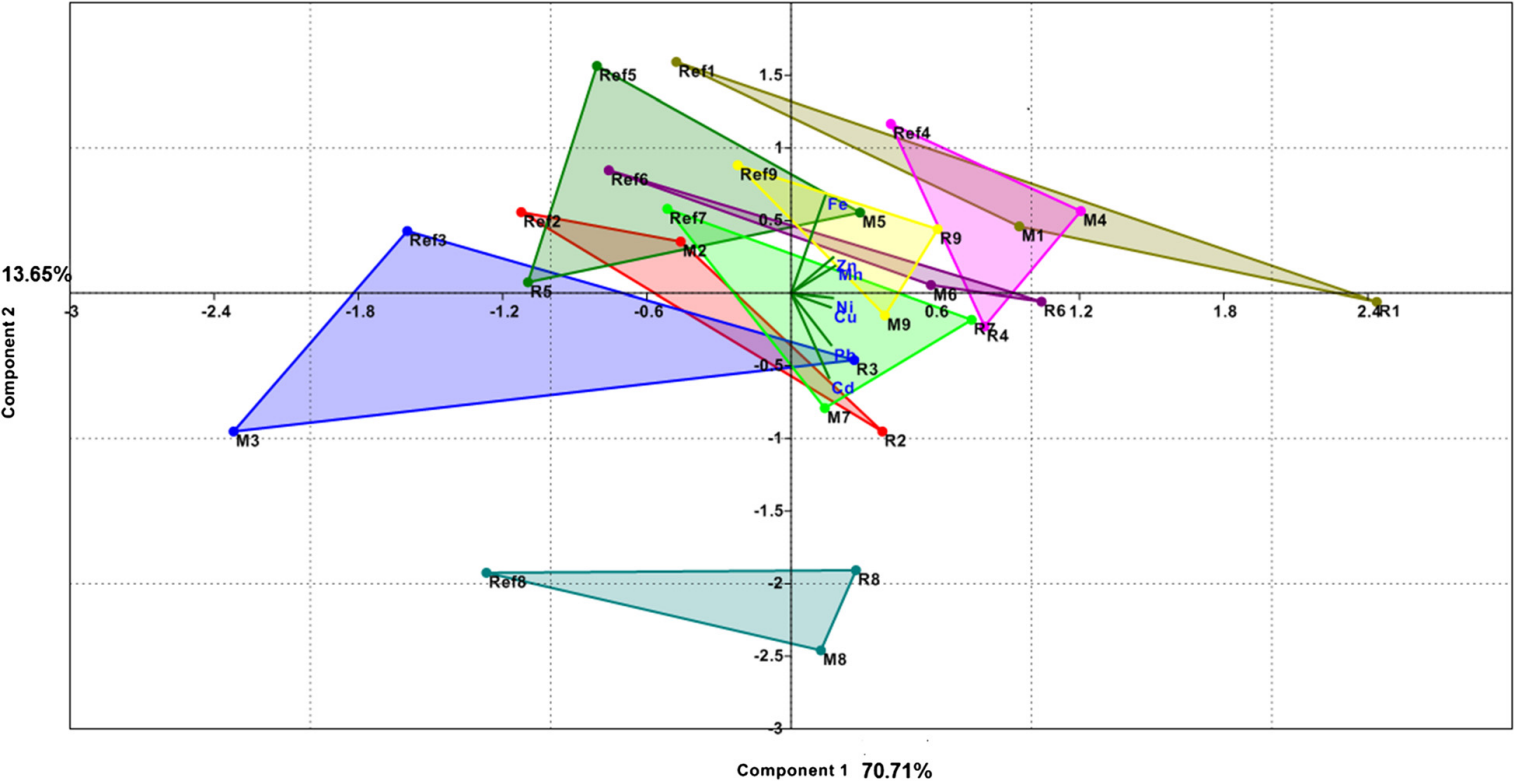
**Biplot of PC1 and PC2 for vegetables data grouped on vegetables species, used PCA3-plant model.** (Fe, Mn, Zn, Cu, Ni, Cd and Pb are variables used in PCA3-plant model that symbolized total contents of metals in vegetable samples, reported to fresh matter; R, M and Ref symbolized vegetables samples from contaminated respectively reference areas and numbers 1 to 9 attached to previous symbols refer to vegetables species or edible part: 1 for parsley roots, 2 for carrot roots, 3 for onion bulbs, 4 for parsley leaf, 5 for carrot leaf, 6 for cabbage, 7 for lettuce, 8 for cucumber and 9 for green bean).

The different accumulation of metals in vegetables can be observed from the results obtained by PCA sorting of vegetable species using PCA3-plant model. This model was computed also only with heavy metals contents of vegetables and its PC1 and PC2 biplot is presented in Figure 5.

The first two PCs explain 85.6% from model variance and can perform a relatively good separation between some vegetable species. Samples of parsley roots (R1, M1) and leaves (R4, M4, Ref4), cabbage (R6, M6), lettuce (R7, M7), green beans (R9, M9) are located on the positive side of PC1, dominated by the presence of Fe, Pb and Cd in samples of the R and M areas and less in samples from Ref area. Samples of roots (M2, Ref2) and leaves of carrot (R5, M5, Ref5) are dominant in the positive side of PC2 and are characterized by high contents of Fe, Zn and Mn. Samples of onions (M3) and cucumbers (Ref8) are predominant in the PC1 and PC2 negative side, and are characterized by low content of heavy metals. Except for parsley leaves (Ref4), all vegetables from Ref area are situated on the negative side of PC1, and are characterized by low content of heavy metals. In this case, supplementation of the PCA3 - plant model with agrochemical parameters (clay content and pH) does not render better ordering of vegetable species (see Additional file 3).

### Principal component analysis of THQ data

Target Hazard Quotients is a complex parameter used for the estimation of potential health risks associated with long term exposure to different pollutants, respectively heavy metals, as in our case. For its calculation, besides the metals content of vegetables, other parameters were also involved, which refer to the metals toxicity (oral reference doses, RfD), the duration and intensity of exposure (exposure frequency, exposure duration, average exposure) and to individual characteristics (average body weight) as well [58-62]. So, THQ is a complex parameter used in health risk assessment of heavy metals which provides a better picture related to the content of metals in soils and vegetables than using a simple parameter [61]. It was developed by the Environmental Protection Agency (EPA) in the US to avoid underestimation of the risk and is calculated by the general formula (1) [58]:

(1) THQ=EFxFDxDIM/RfDxWxT

Where, EF is exposure frequency; FD is the exposure duration, DIM is the daily metal ingestion (mg person^-1^ day^-1^) and RfD is the oral reference dose (mg Kg^-1^ day^-1^); W is the average body weight (Kg) and T is the average exposure time for noncarcinogens (365 days year^-1^× number of exposure years).

A small value of the index (<1) shows reduced health hazard and a value between 1 and 5 represents a concern level for health hazard [58]. THQ parameters used in PCA - THQ model are presented in Table 4. They were computed from data presented in our previous work [61].

**Table 4:** ***THQm *(THQ for males) values for metals of vegetables grown in contaminated and reference areas compiled after Harmanescu et al., 2011 **[61]

**Vegetables and areas**	**Code**	**THQm Fe**	**THQm Mn**	**THQm Zn**	**THQm Cu**	**THQm Ni**	**THQm Cd**	**THQm Pb**	**THQm SUM**
Root parsley R	R1	0.587	0.501	0.283	0.262	0.119	0.365	8.353	**10.47**
Root parsley M	M1	0.262	0.107	0.048	0.319	0.045	0.077	0.350	**1.208**
Root parsley Ref	Ref1	0.129	0.043	0.022	0.057	0.017	0.010	0.040	0.318
Root carrot R	R2	0.079	0.041	0.030	0.071	0.017	0.148	1.117	**1.503**
Root carrot M	M2	0.084	0.029	0.020	0.082	0.007	0.049	0.048	0.319
Root carrot Ref	Ref2	0.046	0.019	0.013	0.034	0.004	0.020	0.029	0.165
Onion R	R3	0.040	0.054	0.067	0.020	0.020	0.105	0.266	0.572
Onion M	M3	0.012	0.018	0.012	0.063	0.001	0.021	0.070	0.197
Onion Ref	Ref3	0.004	0.004	0.005	0.011	0.003	0.010	0.021	0.058
Leaf parsley R	R4	0.092	0.032	0.019	0.027	0.012	0.053	0.347	0.582
Leaf parsley M	M4	0.094	0.043	0.021	0.074	0.058	0.031	0.087	0.408
Leaf parsley Ref	Ref4	0.065	0.026	0.019	0.016	0.009	0.017	0.049	0.201
Leaf carrot R	R5	0.028	0.014	0.008	0.004	0.005	0.007	0.036	0.102
Leaf carrot M	M5	0.045	0.025	0.012	0.033	0.012	0.024	0.022	0.173
Leaf carrot Ref	Ref5	0.013	0.006	0.003	0.006	0.003	0.005	0.005	0.041
Cabbage R	R6	0.159	0.139	0.101	0.063	0.065	0.219	0.474	**1.22**
Cabbage M	M6	0.083	0.121	0.053	0.128	0.031	0.112	0.131	0.659
Cabbage Ref	Ref6	0.042	0.051	0.020	0.021	0.012	0.021	0.028	0.195
Lettuce R	R7	0.095	0.110	0.089	0.086	0.026	0.174	0.326	**0.906**
Lettuce M	M7	0.045	0.054	0.032	0.100	0.017	0.161	0.112	0.521
Lettuce Ref	Ref7	0.036	0.046	0.033	0.035	0.010	0.033	0.040	0.233
Cucumber R	R8	0.006	0.089	0.055	0.045	0.026	0.244	0.379	**0.844**
Cucumber M	M8	0.006	0.079	0.009	0.112	0.050	0.284	0.194	**0.734**
Cucumber Ref	Ref8	0.004	0.006	0.006	0.021	0.021	0.057	0.087	0.202
Green Bean R	R9	0.156	0.093	0.082	0.049	0.045	0.114	0.184	**0.723**
Green Bean M	M9	0.071	0.090	0.063	0.067	0.048	0.123	0.102	0.564
Green Bean Ref	Ref9	0.056	0.059	0.060	0.013	0.020	0.058	0.035	0.301

**Figure 6:**
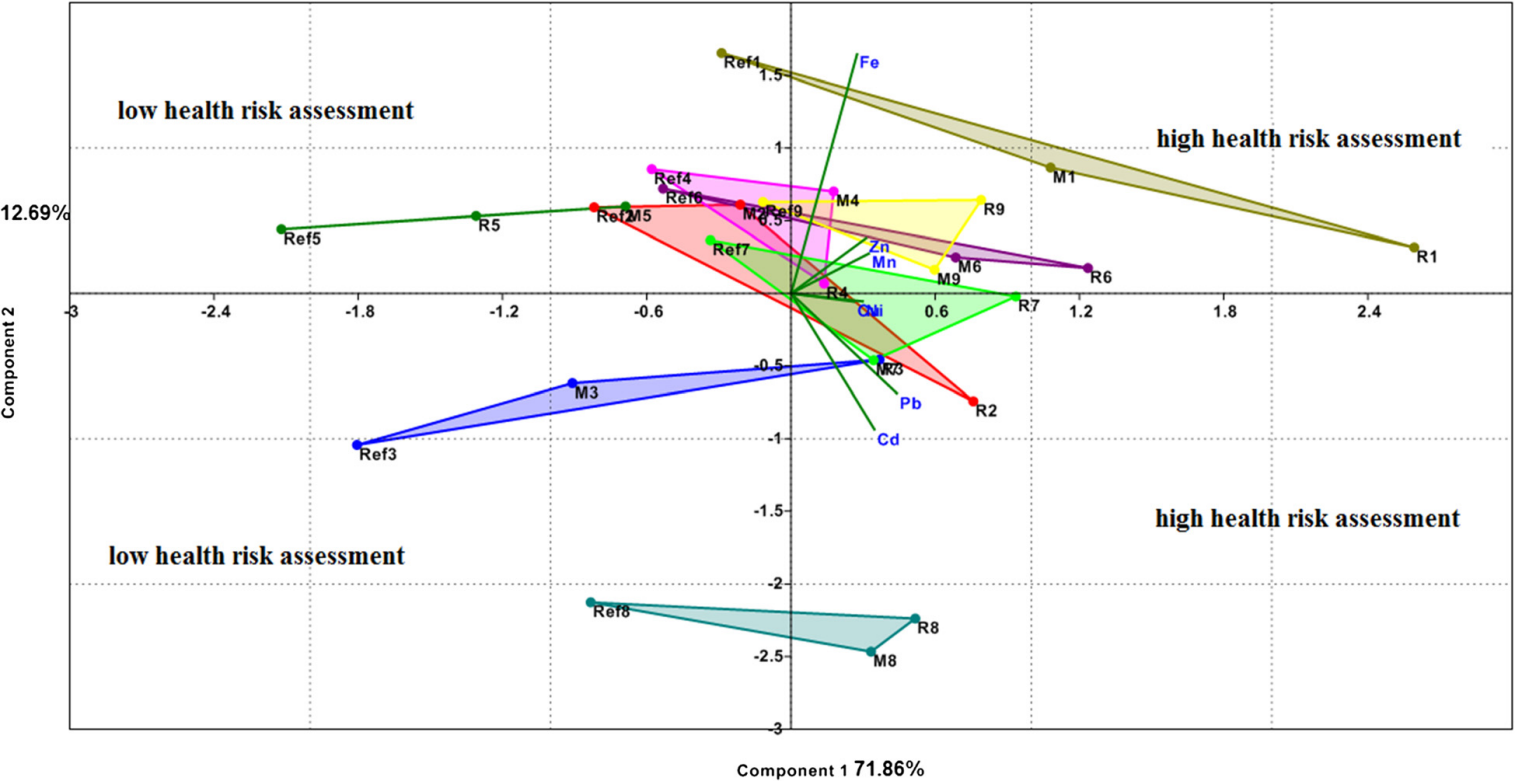
**Biplot of PC1 and PC2 for THQm data grouped on vegetables species, used PCA- THQ model.** (Fe, Mn, Zn, Cu, Ni, Cd and Pb are variables in PCA-THQ model that symbolized THQm for each metals calculated for each vegetable species, compiled after Harmanescu et al., 2011 [61]; R, M and Ref symbolized vegetables samples from contaminated respectively reference areas and numbers 1 to 9 attached to previous symbols refers to vegetables species or edible part: 1 for parsley roots, 2 for carrot roots, 3 for onion bulbs, 4 for parsley leaf, 5 for carrot leaf, 6 for cabbage, 7 for lettuce, 8 for cucumber and 9 for green bean).

Figure 6 presents the results of PCA sorting of vegetables species, applied to THQ data for investigated metals and vegetables.

The first two PCs explain 84.6% from model variance (Figure 6) and can show good separation between some species of vegetables, better than the PCA3 - plant model, which uses as variables only the metals concentrations in vegetables (Figure 5). Better grouping was obtained for parsley roots (R1, M1, Ref1), onion bulbs (R3, M3, Ref3), carrot leaves (R5, M5, Ref5) and cucumber (R8, M8, Ref8) that were well separated from other plants species by PC1 and PC2. From these vegetables, parsley root (R1, M1) is on the PC1 and PC2 positive side, which means that it is associated with the highest THQ values for all metals. In other words, it presents the greatest health risk, especially in contaminated areas R and M. Onion bulbs (M3, Ref3) are on the opposite side, the negative side of the two PCs, which means that they have the lowest THQ values for metals, its lowest health risk respectively, except for contaminated R area. Carrot leaves (R5, M5, Ref5), located on the negative side of PC1 but on the positive side of PC2, are associated with higher THQ values of Fe, Zn and Mn, metals with a low toxicity, so with reduced health risk. Cucumbers (R8, M8), located on the positive side of PC1 and on the negative side of PC2, are associated with higher Cd and Pb concentrations and THQ values for these toxic metals, so with increased health risk, especially in contaminated R and M areas. The other plants, carrot roots (R2, M2, Ref2), parsley leaves (R4, M4, Ref4), cabbage (R6, M6, Ref6), lettuce (R7, M7, Ref7) and green beans (R9, M9, Ref9 ) are not satisfactorily separated, being merged in the centre of the axes. These vegetables grown in contaminated R and M areas are associated with high THQ values, especially for toxic metals Cd and Pb, which mean that their consumption presents a major health risk. In the reference area, these plants are associated with low levels of THQ, so the health risk linked to their consumption is low.

## Conclusions

This legacy of heavy metals pollution generated by industrial society put pressure on human health all over the world. Finding a solution for this situation is a permanent task of researchers, which involves not only finding new and advanced analytical methods to identify quality and quantity of contaminants, but also applying complex statistical methods that allow an overall assessment of the interaction of these contaminants in the food chain and the health risk associated with their consumption by humans. In our study, application of PCA for analysing these complex data provides:

– optimization of analytical procedures by selecting for analysis only 4 variables (Cu, Zn, Cd and Pb) with maximum involvement in pollution assessment, and thus reduction of the analytical costs;

– differentiation among contaminated areas and types of contamination, based on soil analyses; in this case the emphasis of the pollution with Pb in R zone and with Cu in M zone;

– selection of vegetables species which the highest (parsley roots, lettuce, beans and cucumbers) or lowest (onions, carrot leaves) health risk in contaminated areas;

– selection of the best markers which can establish the foods with the highest/ lowest risk on human health in affected areas; in this case the complex THQ parameter permits a better classification of the vegetables with high risk of toxicity for the human health produced by the heavy metals;

– building and viewing models that can make it easier to understand the complex phenomenon of environmental pollution and health risk.

### Experimental

Detailed description of the location of investigated sites, the preparation of soil and vegetable samples, the analysis of heavy metals and quality control are presented in our previous work [61]. Briefly, the study areas are located in the South West of Romania, in Banat County (see Experimental site location in the Additional file 4). Soil and vegetable samples were collected from subsistence farms located in the two contaminated areas (R and M) and one reference area (Ref). The first contaminated area (R) is located around Ruschita village which is the mining centre of Poiana Rusca Mountain. In this area, soil has a gritty texture, the clay content is between 18-22% and the pH is near to 7.8 (neutral) [63]. The second contaminated area (M) is located around Moldova Noua town, close to the Danube river. Soil from this area also has a gritty texture, clay contents between 18-20% and a pH near to 7.6 (neutral) [63]. The reference area, Borlova village, near the town of Caransebes, is located on the Sebes river valley, at the foot of Muntele Mic Mountain, a non-polluted area with less industry. Soil from this area has a fine texture, clay content between 28-32% and a pH near to 6.6 (slightly acid) [63]. From each area (R, M and Ref) were collected 9 average soil samples, from 0 to 20 cm deep. Average soil samples were prepared from 10 individual soil samples.

The extraction of HMs from soil samples were made by the wet procedure with a mixture of mineral acids (HCl, HNO_3_, 3:1 ratio) and from vegetables by plant ash digestion with 0.5N HNO_3_. The plant ash was obtained by burning plant samples 8 h at 550°C in the furnace (Nabertherm B150, Lilienthal, Germany). HMs were analysed from solutions by flame atomic absorption spectrometry (FAAS) in University Environmental Research Test Laboratory using the flame atomic absorption spectrophotometer with high resolution continuum source (Model ContrAA 300, Analytik Jena,Germany), fitted with a specific condition for particular metals using appropriate drift blanks. NCS Certified Reference Material-DC 85104a and 85105a (China National Analysis Center for Iron&Steel), were analysed for quality assurance. Per cent recovery means were: Fe (92%), Mn (95%), Zn (102%), Cu (105%), Ni (99%), Pb (94%), Cd (105%). The variation coefficients were below 10%. Detection limits (μg/g) were determined by the calibration curve method: Fe (0.15), Mn (0.19), Zn (0.43), Cu (0.13), Ni (0.14), Cd (0.01), Pb (0.05) [61].

The levels of heavy metals for soil samples from these areas are presented in Table 1 and the data for average HMs contents in vegetables, compiled according to Harmanescu et al., 2011 [61], in Table 3.

### Statistics

The data were statistically analysed using a statistical package PAST [42]. The concentrations of metals contents were expressed in terms of means and standard deviation, and the figures with the mean values. Statistical significance was computed using Pair-Samples T-Test, with a significance level of **p.**

## Competing interests

The authors declare that they have no competing interests.

## Authors’ contributions

The two authors have had equal contribution to realize all the steps of this scientific paper. IG and MH read and approved the final manuscript. All authors read and approved the final manuscript.

## Supplementary Material

Additional file 1: Figure 2A Biplot of PC1 and PC2 for soil data in PCA2-soil model. (ZnS, CuS, CdS and PbS are variables used in PCA1-soil model that symbolized total content of Zn, Cu, Cd and Pb in soil samples; R, M and Ref symbolized soils samples from contaminated respectively reference areas and numbers 1 to 9 attached to previous symbols refer to soils samples associated with vegetables species or edible part: 1 for parsley roots, 2 for carrot roots, 3 for onion bulbs, 4 for parsley leaf, 5 for carrot leaf, 6 for cabbage, 7 for lettuce, 8 for cucumber and 9 for green bean). Click here for file

Additional file 2: Figure 4A Biplot of PC1 and PC2 for vegetable data grouped on location, used PCA1-plant model. (FeP, MnP, ZnP, CuP, NiP, CdP and PbP are variables used in PCA1-plant model that symbolized total contents of Fe, Mn, Zn, Cu, Ni, Cd and Pb in vegetable samples, reported to fresh matter; R, M and Ref symbolized vegetables samples from contaminated respectively reference areas and numbers 1 to 9 attached to previous symbols refer to vegetables species or edible part: 1 for parsley roots, 2 for carrot roots, 3 for onion bulbs, 4 for parsley leaf, 5 for carrot leaf, 6 for cabbage, 7 for lettuce, 8 for cucumber and 9 for green bean). Click here for file

Additional file 3: Figure 5A Biplot of PC1 and PC2 for vegetables data grouped on vegetables species, used PCA3-plant model supplemented with agrochemical parameters pH and clay content. Click here for file

Additional file 4 Experimental site location. Click here for file
