# Supplementary figures and images for: Application of principal component analysis in the pollution assessment with heavy metals of vegetable food chain in the old mining areas
Source: Chem Cent J. 2012 Dec 13;6:156. doi: 10.1186/1752-153X-6-156 (PMC3575243; doi:10.1186/1752-153X-6-156)

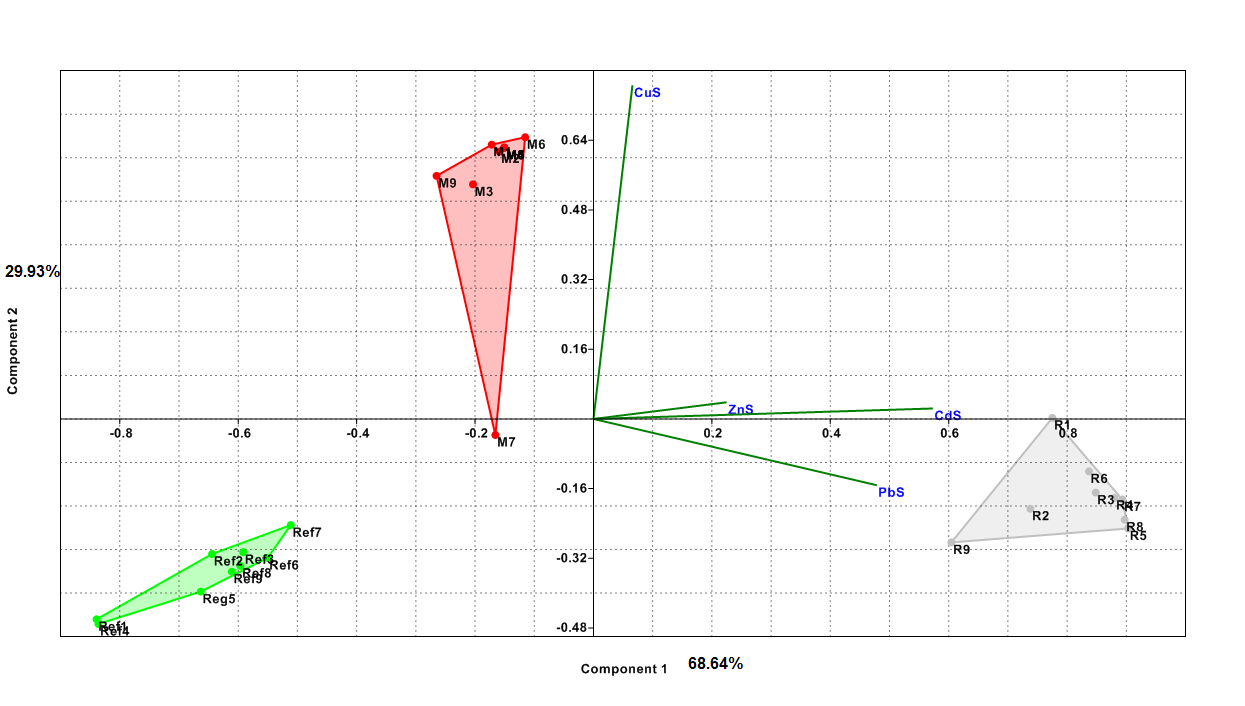

Supplement: Additional file 1: Figure 2A — Biplot of PC1 and PC2 for soil data in PCA2-soil model. (ZnS, CuS, CdS and PbS are variables used in PCA1-soil model that symbolized total content of Zn, Cu, Cd and Pb in soil samples; R, M and Ref symbolized soils samples from contaminated respectively reference areas and numbers 1 to 9 attached to previous symbols refer to soils samples associated with vegetables species or edible part: 1 for parsley roots, 2 for carrot roots, 3 for onion bulbs, 4 for parsley leaf, 5 for carrot leaf, 6 for cabbage, 7 for lettuce, 8 for cucumber and 9 for green bean). [file 1752-153X-6-156-S1.png]

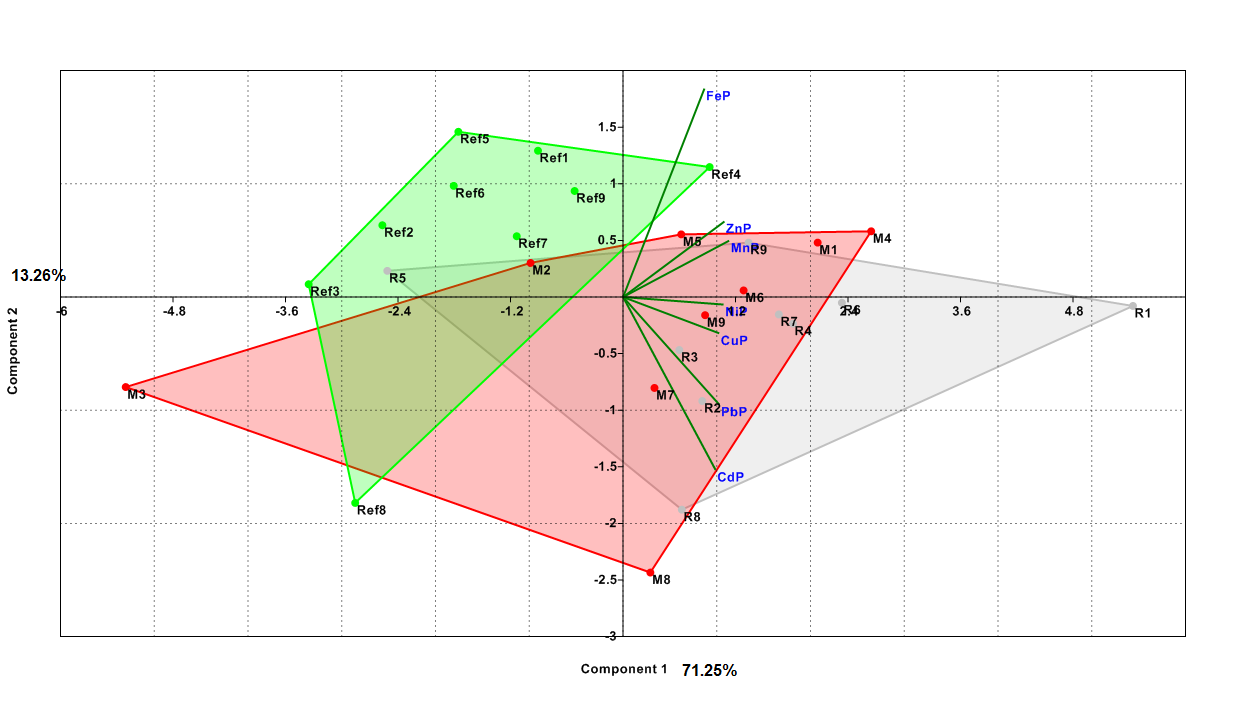

Supplement: Additional file 2: Figure 4A — Biplot of PC1 and PC2 for vegetable data grouped on location, used PCA1-plant model. (FeP, MnP, ZnP, CuP, NiP, CdP and PbP are variables used in PCA1-plant model that symbolized total contents of Fe, Mn, Zn, Cu, Ni, Cd and Pb in vegetable samples, reported to fresh matter; R, M and Ref symbolized vegetables samples from contaminated respectively reference areas and numbers 1 to 9 attached to previous symbols refer to vegetables species or edible part: 1 for parsley roots, 2 for carrot roots, 3 for onion bulbs, 4 for parsley leaf, 5 for carrot leaf, 6 for cabbage, 7 for lettuce, 8 for cucumber and 9 for green bean). [file 1752-153X-6-156-S2.png]

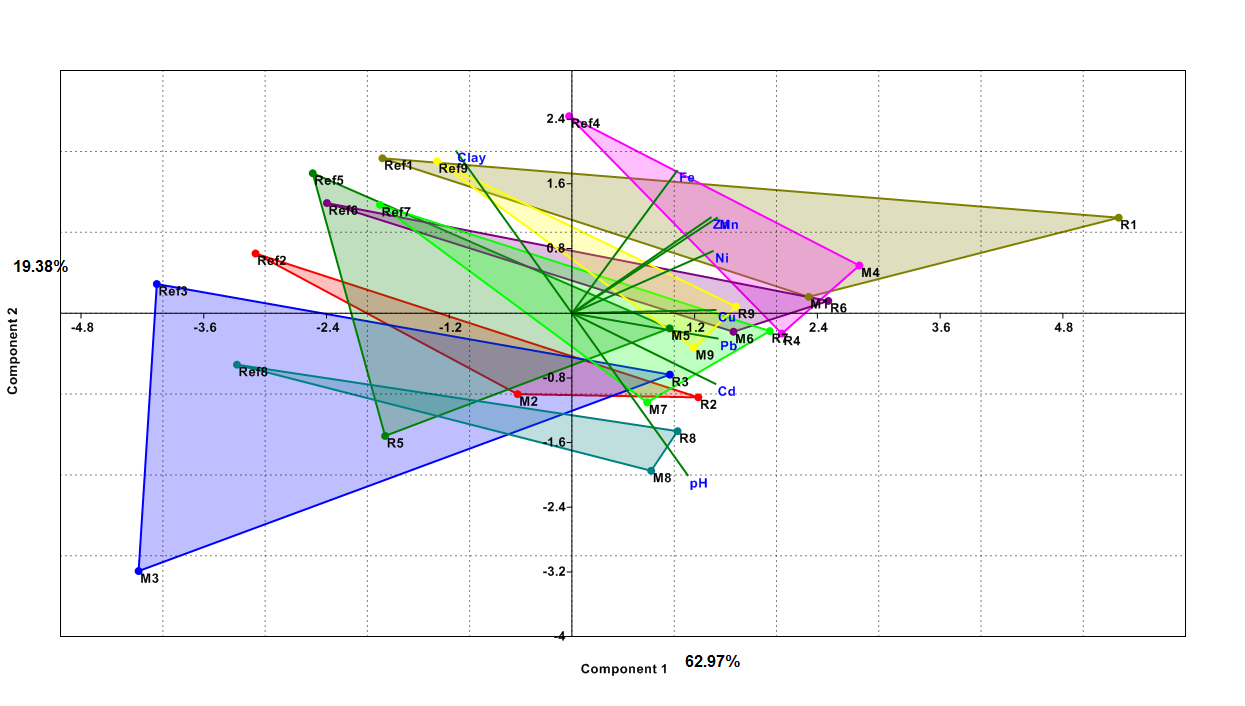

Supplement: Additional file 3: Figure 5A — Biplot of PC1 and PC2 for vegetables data grouped on vegetables species, used PCA3-plant model supplemented with agrochemical parameters pH and clay content. [file 1752-153X-6-156-S3.png]

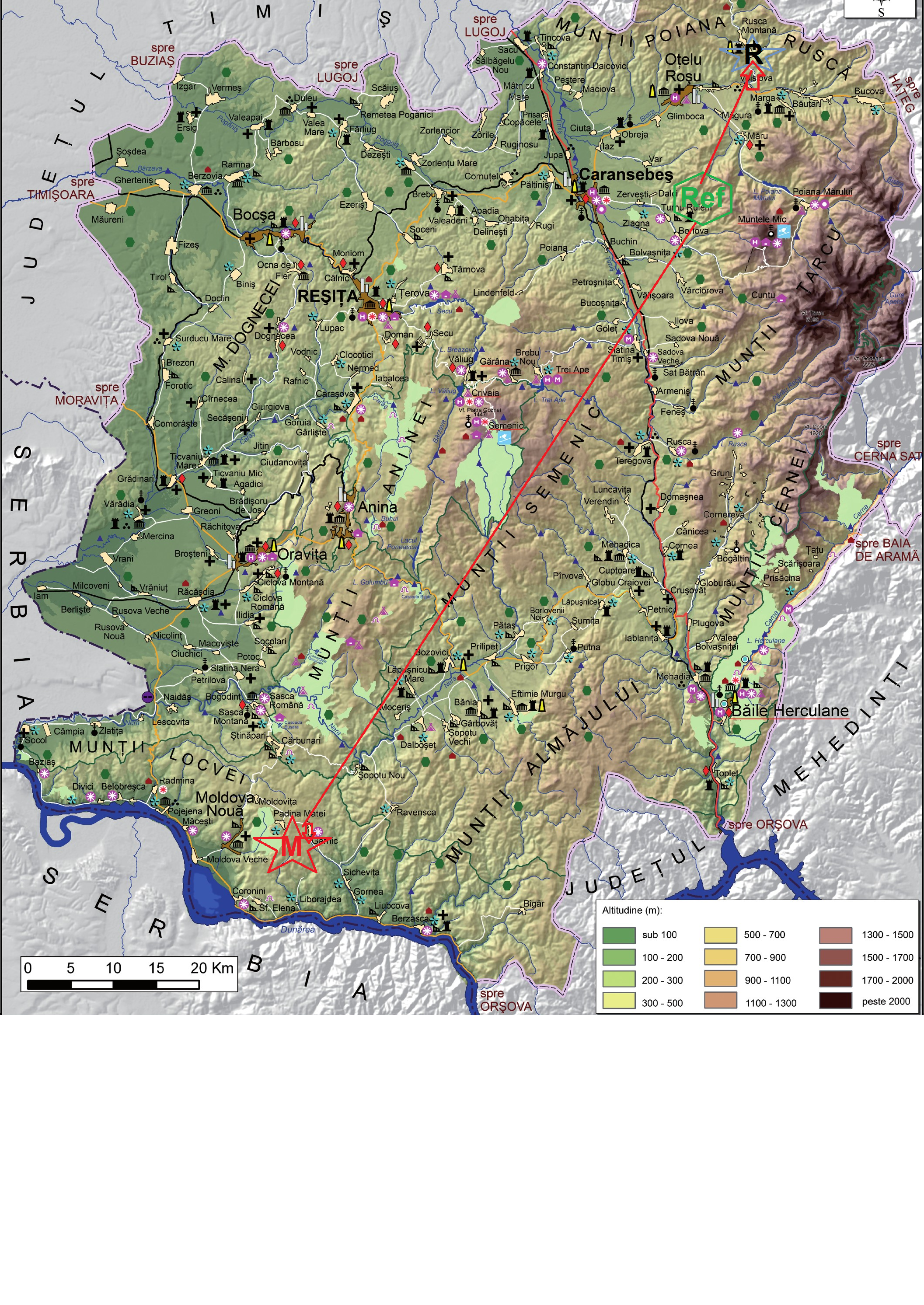

Supplement: Additional file 4 — Experimental site location. [file 1752-153X-6-156-S4.png]
